# Solitary Plasmacytoma of the Cecum and the Ascending Colon: Surgical Resection as a Treatment Modality

**DOI:** 10.1155/2015/126863

**Published:** 2015-04-12

**Authors:** Tahsin Dalgic, Erdal Birol Bostanci, Tebessum Cakir, Ilter Ozer, Murat Ulas, Gulden Aydog, Musa Akoglu

**Affiliations:** ^1^Department of Gastroenterological Surgery, Turkiye Yuksek Ihtisas Training and Research Hospital, 06230 Ankara, Turkey; ^2^Department of Pathology, Turkiye Yuksek Ihtisas Training and Research Hospital, 06230 Ankara, Turkey

## Abstract

Colonic solitary plasmacytoma is a rare disease, with few reports occurring in the literature. Solitary plasmacytoma is defined as a plasma cell tumour with no evidence of bone marrow infiltration. Plasmacytoma can present as a solitary tumour in bone or in other parts of the body. The gastrointestinal tract is rarely the site of the disease. We report on the case of a 51-year-old man presenting with a colonic symptomatic mass with unclear biopsy results. A resected specimen showed a solitary plasmacytoma. Surgical resection was an adequate treatment modality in this case. Endoscopic resection, radiotherapy, and chemotherapy are also preferred treatments in selected gastrointestinal plasmacytoma cases.

## 1. Introduction

Solitary plasmacytoma (SP), a monoclonal proliferation of plasma cells without evidence of significant bone marrow plasma cell infiltration, occurs in two main forms: bone SP (P-bone) and extramedullary SP (P-extramedullary). SP is a rare disease and accounts for about 10% of plasma cell tumours [1]. P-extramedullaries are most often located in the head and neck region, mainly in the upper aerodigestive tract, but may also occur in the gastrointestinal (GI) tract, urinary bladder, central nervous system (CNS), thyroid, breast, testes, parotid gland, lymph nodes, and skin. About 10% of P-extramedullaries occur in the GI tract [2]. Involvement of the colon is extremely rare, with only 32 cases reported in the literature [3]. This study reports on the case of a colonic mass presenting as a colonic tumour.

## 2. Case Report

**Figure 1:**
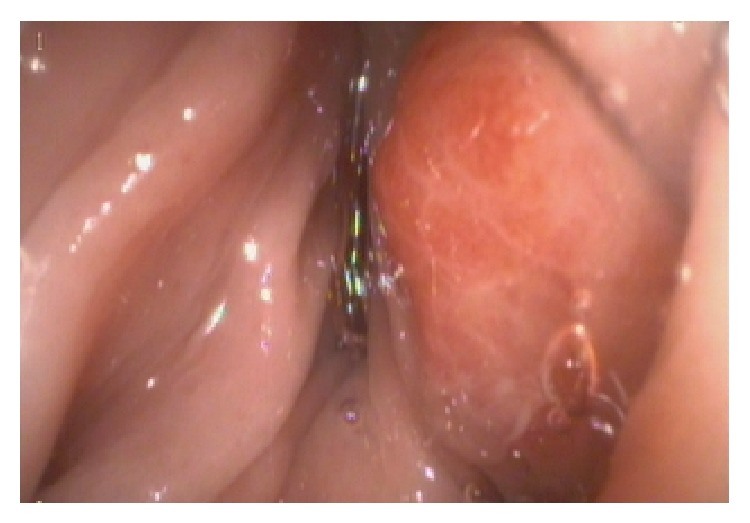
Colonoscopic appearance of lesion.

**Figure 2:**
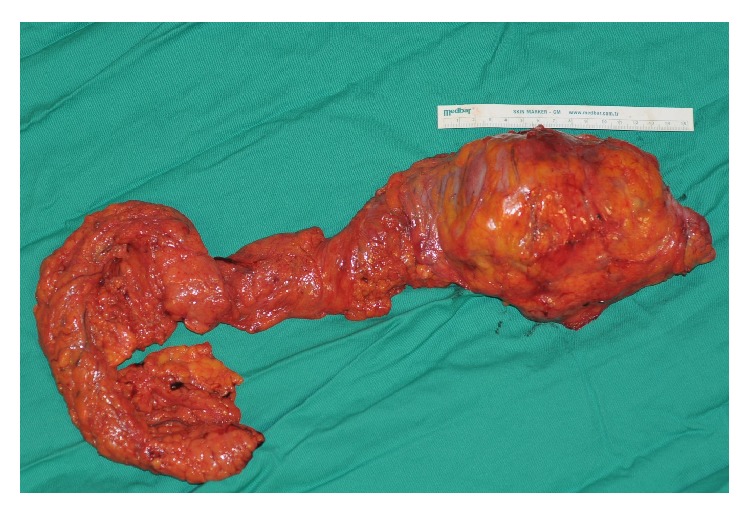
Resected specimen.

A 51-year-old man presented with 6 months' history of abdominal pain, changes in bowel habits, bloody diarrhoea, and a 10 kg loss of weight. No palpable mass was found in the abdomen during physical examination. Liver and kidney function tests, the carcinoembryonic antigen (CEA) level, and the complete blood count were normal except for a measured haemoglobin (Hb) level of 9.3 gr/dL. His ultrasonography (USG) examination showed a 9.5 cm long annular mass at the cecum and the ascending colon. An abdominal computed tomography (CT) scan showed a 3 cm wide and 10 cm long annular mass with medial extension into the fatty tissue at the same localization. The colonoscopy revealed a constricting ulcerovegetative mass localized at the medial border of the cecum in close proximity to the ileocecal valve extending into the ascending colon (Figure 1). A biopsy of the lesion showed atypical cells with hyperchromatic pleomorphic nuclei characteristic of an undifferentiated tumour but also resembling mononuclear cells associated with inflammatory bowel disease. The colonoscopy was repeated, and a rebiopsy showed an infiltration of plasmacytoid mononuclear cells with Kappa monoclonality. Serum protein electrophoresis results were normal, and a test for Bence-Jones protein in urine was negative. A bone scintigraphy revealed no abnormality other than a hemangioma in one of the vertebrae. After the exclusion of disseminated plasma cell malignancies, a right hemicolectomy with ileotransversostomy was performed (Figure 2). The patient was discharged at postoperative day 7 without any morbidity.

**Figure 3:**
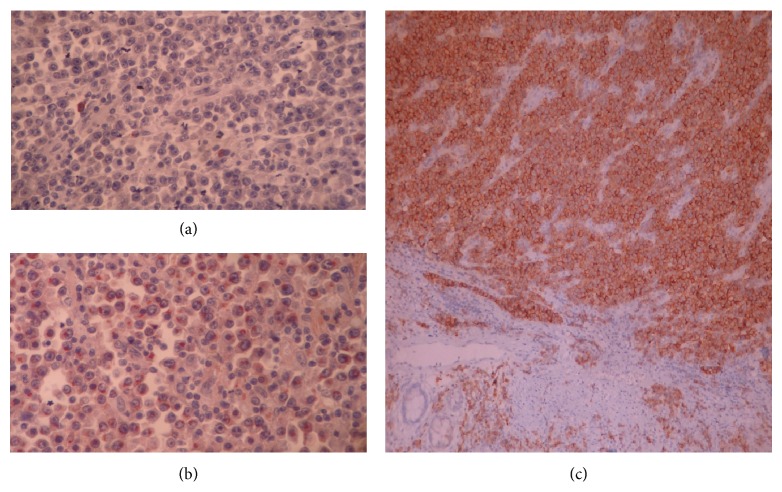
Many of the plasma cells reacted with antibody to Kappa light chain (b) and were not stained with antibody to Lambda light chain except for fewer nonneoplastic plasma cells (a). The atypical plasmocytes expressed surface CD 38 (c).

Histopathological examination of the resected colon showed atypical plasmacytoid cells staining Kappa light chain positive and Lambda light chain negative. The Ki-67 proliferation index was 40%. CD43, CD3, and CD20 staining were negative, while that for CD38 was positive. The tumour was reported as a malign plasma-cell neoplasia (Figure 3). None of the isolated lymph nodes showed plasmacytic cell infiltration. The patient did not receive any other treatment besides surgery and was followed up for three years with no recurrence, distant disease, or multiple myeloma (MM) progression.

## 3. Discussion

Plasmacytomas, first described by Schridde in 1905 [4], are clonal proliferations of plasma cells that are cytologically and immunophenotypically identical to plasma cell myeloma but manifest a localized osseous or extraosseous growth pattern. They are rare diseases, and our understanding of their epidemiologic features and clinical outcomes is largely derived from compilations of cases reported in the literature and in small clinical series [5].

The incidence of P-bone is approximately 40% higher than that of P-extramedullary. The male-to-female ratio is somewhat higher for P-extramedullary than for P-bone. Nearly 30% of P-extramedullaries are of respiratory origin, and 24% occur in the mouth orthopharynx. P-extramedullaries rarely occur in the GI tract, stomach, small bowel, colon, or rectum. SP usually presents in the 5th or 6th decades [6].

Diagnostic criteria for SP have varied over time. Both P-extramedullary and P-bone should be basically associated with a normal bone marrow [6]. All of the following four criteria must be met: a biopsy-proven solitary lesion of bone or soft tissue with evidence of clonal plasma cells; normal bone marrow with no evidence of clonal plasma cells; a normal skeletal survey; and the absence of end-organ damage [7]. Most authors agree that the detection of a monoclonal band on serum protein electrophoresis or Bence-Jones protein in urine does not necessarily preclude the diagnosis. It is estimated that about 25% of EMP will show a monoclonal band of serum protein at the time of diagnosis. The monoclonal gammopathy disappears following treatment of the localized primary tumour [8].

The presentation of gastrointestinal plasmacytoma (P-GI) is different from that occurring at other sites in the body. The most common presenting symptom is abdominal pain. Other symptoms can include rectal bleeding, a change in bowel habits, weight loss, nausea, vomiting, large bowel obstruction, and intussusception [1]. P-GIs are generally visible as well-defined soft-tissue masses on a CT scan and have heterogeneous enhancement on a magnetic resonance imaging (MRI) scan. Larger plasmacytomas can show aggressive traits such as the invasion of adjacent fat, bone erosion, or vascular encasement. As well, 18F-FDG PET/CT has been found to be useful in staging and follow-up of P-extramedullary [3].

In general, P-extramedullaries are considered to be radiosensitive, with a local control rate of 90–100% [5]. Allison et al. [9] propose surgery, radiotherapy (RT), and chemotherapy for P-GI according to the degree of disease extension. Colonic plasmacytoma (C-P) occurs only rarely, and a definitive algorithm for treatment has not been determined. Endoscopic treatments such as submucosal resection or polypectomy have proven to be sufficient in selected cases [10–12]. RT is the preferred treatment for anal canal and rectal lesions [2, 13]. An extensive review of over 400 publications done by Alexiou et al. provides evidence that surgery alone gives the best results in cases of SP when resectability is good. However, if complete surgical tumour resection is doubtful or impossible and/or if lymph node areas are affected, then combined therapy (surgery and radiation) is recommended [14]. In one recent series with 80 patients, younger patients, especially those with head-neck lesions and without pre-RT macroscopic tumours, seem to have the best outcome when treated with RT, either with or without surgery [15].

In only one randomised trial of chemotherapy, patients receiving melphalan and prednisone showed an improved disease-free survival rate [16]. Susnerwala et al. have proposed a pathological grading system for S-P based on the MM grading criteria, with tumours classified into low, intermediate, and high grades, and this system has been found to correlate closely with outcomes [8]. The use of adjuvant chemotherapy is recommended for patients with higher-grade disease, local treatment failure (tumour size of >5 cm), or refractory disease [5].

P-extramedullary patients characteristically present with localized disease, and the incidence of lymph node involvement is 10–20%. The overall 10-year survival rate is ~70% [8]. Survival rates range from more than 90% among patients with P-extramedullary arising in the skin or lymph nodes to 48% for those with eye/brain/CNS tumours [6]. Progression to MM varies from 10% to 30% in P-extramedullary. In a series with 258 SP patients (52 with P-extramedullary), bone localization was found to be the only predictor of MM development on multivariate analyses [17]. When progression to MM occurs, it usually does so within the first 2 years [8]. Follow-up radiological and electrophoresis assessment is required following treatment to detect recurrence and progression to MM.

P-extramedullaries are rarely seen in the GI tract. It is important to consider this possibility during the evaluation of a mass in the GI tract because P-GIs have different treatment and follow-up modalities than adenocarcinomas and other tumours of GI. Surgical treatment is usually sufficient in localized P-C. In our case, with localized disease and no lymph node involvement, there has been no recurrence or MM progression in three years.
